# Supplementary material for: Reef islands have continually adjusted to environmental change over the past two millennia
Source: Nat Commun. 2023 Jan 31;14:508. doi: 10.1038/s41467-023-36171-2 (PMC9889315; doi:10.1038/s41467-023-36171-2)
Supplement: Supplementary file 1 — Supplementary Information [file 41467_2023_36171_MOESM1_ESM.pdf]

Supplementary Information

Reef islands have continually adjusted to environmental change over the past two millennia

Kench, P.S., Liang, C., Ford, M.R., Owen, S.D., Mohamed, A., Ryan, E.J., Turner, T., Beetham, E., Dickson, M.E., Stephenson, W., Vila-Concejo, A., McLean, R.F.

List of Supplementary Figures and Tables

Supplementary Figure 1. Location of the Kandahalagalaa field site.

Supplementary Figure 2. Kandahalagalaa reef platform and island.

Supplementary Figure 3. Summary of the frequency distribution of radiometric ages on sediments and beachrock samples from the Kandahalagalaa reef platform and island.

Supplementary Table 1. Source references of island evolution studies presented in Figure 1B.

Supplementary Table 2. Radiocarbon ages, Kandahalagalaa, Huvadhoo atoll, Maldives.

Supplementary Table 3. Physical properties of beach rock outcrops on Kandahalagalaa, Huvadhoo Atoll, Maldives.

Supplementary Table 4. Calculated vegetated area of Kandahalagalaa Island, Huvadhoo atoll, 1969-2021 for the aerial images used in the analysis.

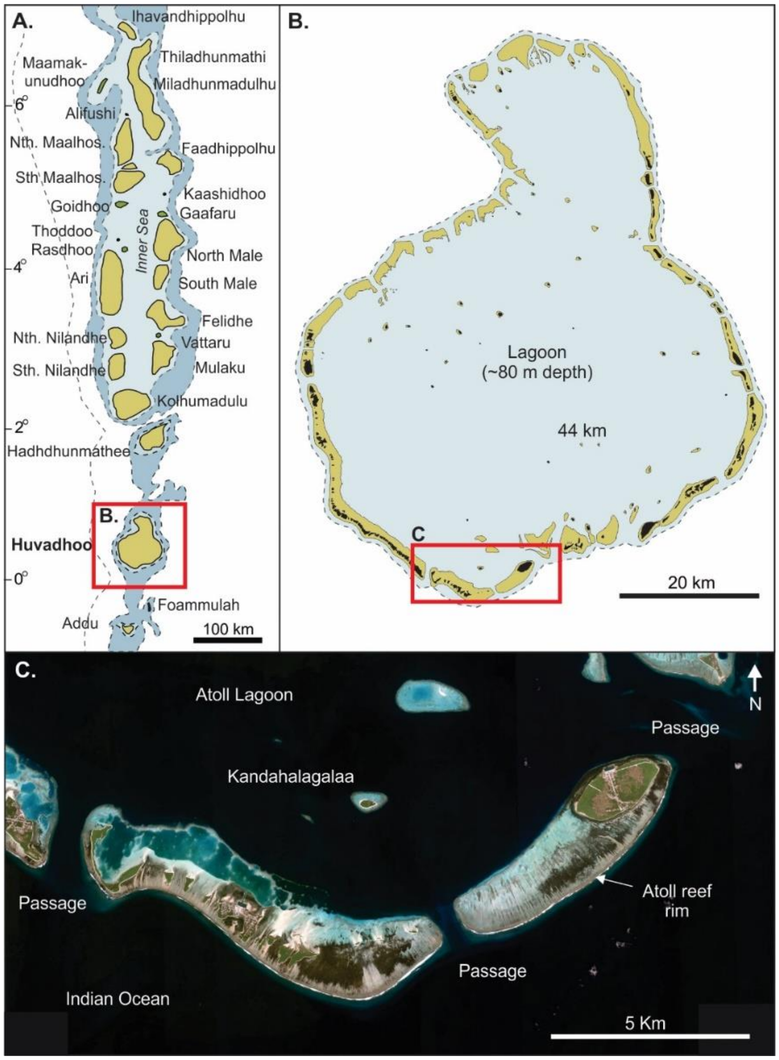

Supplementary Figure 1. Location of the Kandahalagalaa field site. A) Maldives archipelago in the central Indian Ocean. B) Huvadhoo atoll, southern Maldives. C) Southwest sector of Huvadhoo atoll showing the outer atoll reef rim and location of the Kandahalagalaa reef platform. Satellite image is from Google Earth, Maxar Technologies, Image Landsat/Copernicus taken 2015. Map data © 2022 Google.

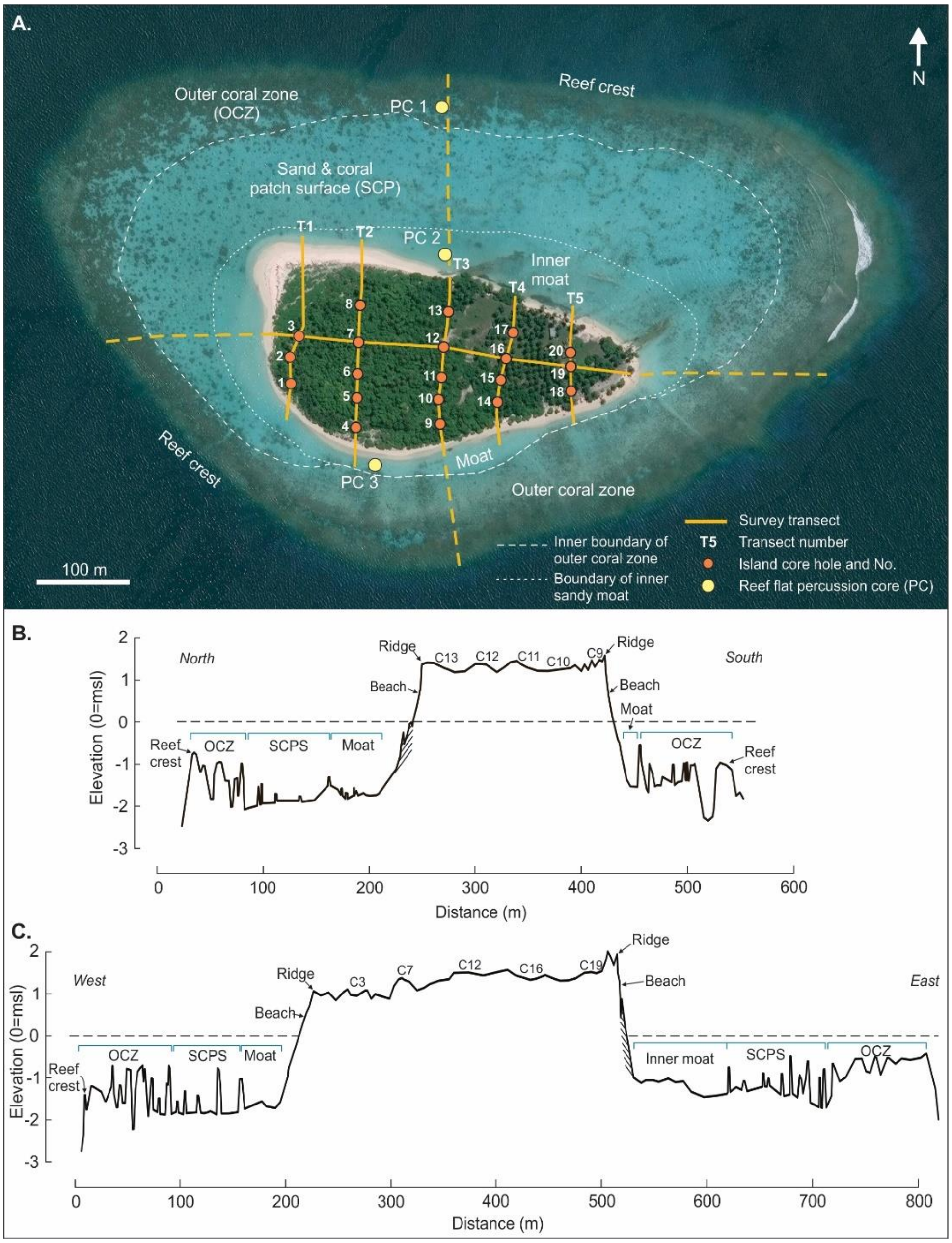

Supplementary Figure 2. Kandahalagalaa reef platform and island. A) Summary of major eco-physiographic zones and field sampling sites on the Kandahalagalaa reef platform and island. Source: Google image. B and C) topographic surveys across the reef platform and island surface. Satellite image © 2019 Maxar Technologies.

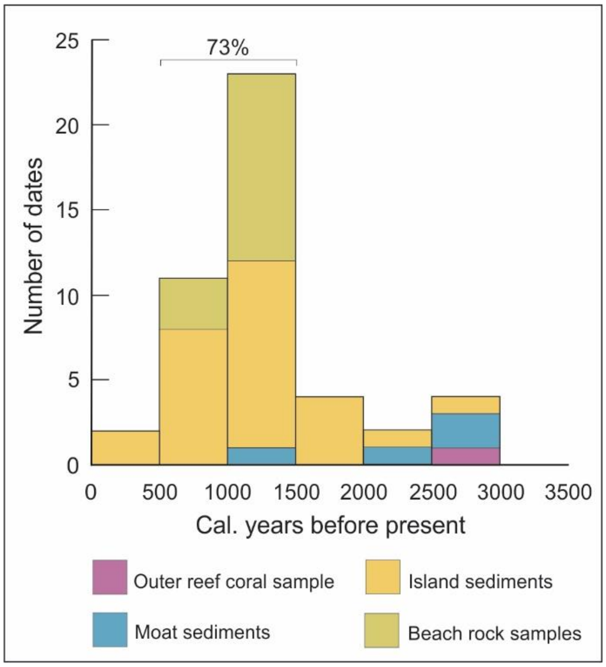

Supplementary Figure 3. Summary of the frequency distribution of radiometric ages on sediments and beachrock samples from the Kandahalagalaa reef platform and island. All radiometric dates are presented in Supplementary Figure 1.

Supplementary Table 1. Source references of island evolution studies presented in Figure 1B.

Island	Atoll	Country	Reference
a. Mainadhoo	Huvadhoo	Maldives	East, H. et al. 2018. Coral reef island initiation and development under higher than present sea levels. *Geophysical Research Letters,* 45, doi.org/10.1029/2018GLO79589.
b. Boduhini,	Huvadhoo	Maldives	East, H. et al. 2018. Coral reef island initiation and development under higher than present sea levels. *Geophysical Research Letters,* 45, doi.org/10.1029/2018GLO79589.
c. Galamadhoo	Huvadhoo	Maldives	East, H. et al. 2018. Coral reef island initiation and development under higher than present sea levels. *Geophysical Research Letters,* 45, doi.org/10.1029/2018GLO79589.
d. Baavanadhoo	Huvadhoo	Maldives	East, H. et al. 2018. Coral reef island initiation and development under higher than present sea levels. *Geophysical Research Letters,* 45, doi.org/10.1029/2018GLO79589.
e. Kandahalagalaa	Huvadhoo	Maldives	Liang, Y. et al. 2022. Lagoonal reef island formation in Huvadhoo atoll, Maldives, highlights marked temporal variations in island building across the archipelago. *Geomorphology*. 10.1016/j.geomorph.2022.108395
f. Kondey	Huvadhoo	Maldives	Liang, Y. et al. 2022. Lagoonal reef island formation in Huvadhoo atoll, Maldives, highlights marked temporal variations in island building across the archipelago. *Geomorphology*. 10.1016/j.geomorph.2022.108395
g. Vaadhoo	Huvadhoo	Maldives	Kench, P.S. et al. 2020. Holocene sea level dynamics drive formation of a large atoll island in the central Indian Ocean. *Global and Planetary Change*, 195, 103354.
h. Dhakandhoo	South Maalhosmadulu	Maldives	Kench, P.S. et al. 2005. New model of reef-island evolution: Maldives, Indian Ocean. *Geology*, 33, 145-148.
i. Hulhudhoo	South Maalhosmadulu	Maldives	Kench, P.S. et al. 2005. New model of reef-island evolution: Maldives, Indian Ocean. *Geology*, 33, 145-148.
j. Thiladhoo	South Maalhosmadulu	Maldives	Kench, P.S. et al. 2005. New model of reef-island evolution: Maldives, Indian Ocean. *Geology*, 33, 145-148.
k. Cocos	Cocos	Australia	Woodroffe C. et al., 1999 Marine Geology
l. Warraber	Torres Strait	Australia	Woodroffe, C.D. et al. 2007. Incremental accretion of a sandy reef island over the past 3000 years indicated by component-specific radiocarbon dating. *Geophysical Research Letters* 34, L03602.
m. Bewick	Great Barrier Reef	Australia	Kench, P.S., et al. 2012. Rapid reef island formation and stability over an emerging reef flat: Bewick Cay, northern Great Barrier Reef, Australia. *Geology*, 40, 347–350. doi: 10.1130/G32816.1
n. Lady Elliot	Great Barrier Reef	Australia	Chivas A. et al., 1986. Radiocarbon evidence for the timing and rate of Island development, beach-rock formation and phosphatization at Lady Elliot Island, Queensland, Australia. *Marine Geology,* 69, 273-287.
o. Mba	-	New Caledonia	Yamano, H. 2014. Late Holocene sea-level change and reef-island evolution in New Caledonia. *Geomorphology*, 222, 39-45.
p. Tepuka	Funafuti	Tuvalu	Kench, P.S., et al. 2014. The geomorphology, development and temporal dynamics of Tepuka Island, Funafuti Atoll, Tuvalu. *Geomorphology.*, 222, 46-58.
q. Tutaga	Funafuti	Tuvalu	Kench, P.S. 2018. Storm-deposited coral reef blocks: a mechanism of island genesis, Tutaga, Funafuti, Tuvalu. *Geology*, 46, 915-918.
r. Laura	Majuro	Marshall Islands	Kayanne, H. et al. 2011. Rapid settlement of Majuro Atoll, central Pacific, following its emergence at 2000 years CalBP. *Geophysical Research Letters* 38, no.20.
s. Jabat	Jabat	Marshall Islands	Kench, P.S. et al. 2014. Evidence for Coral Island Formation During Rising Sea Level in the Central Pacific Ocean. *Geophysical Research Letters*, 41, doi:10.1002/2013GL059000.
t. Jeh	Jeh	Marshall Islands	Ford, M. et al., 2020. Active sediment generation on coral reef flats contributes to recent island expansion. *Geophysical Research Letters*, 47, e2020GL088752
u. Jabnodren	Jaluit	Marshall Islands	Kench, P.S., et al. 2022. Heightened storm activity drives late Holocene reef island formation in the central Pacific Ocean. *Global and Planetary Change*, 215, 103, 103888. https://doi.org/10.1016/j.gloplacha.2022.103888
v. Jin	Jaluit	Marshall Islands	Kench, P.S., et al. 2022. Heightened storm activity drives late Holocene reef island formation in the central Pacific Ocean. *Global and Planetary Change*, 215, 103, 103888. https://doi.org/10.1016/j.gloplacha.2022.103888
w. Malamala	Nadi Bay	Fiji	McKoy, H. et al. 2010. Sand cay evolution on reef platforms, Mamanuca Islands, Fiji. *Marine Geology,* 269, 61-73.
x. Navini	Nadi Bay	Fiji	McKoy, H. et al. 2010. Sand cay evolution on reef platforms, Mamanuca Islands, Fiji. *Marine Geology,* 269, 61-73.
y. Makin	Makin	Kiribati	Woodroffe C. & Morrison 2001. Reef-island accretion and soil development on Makin, Kiribati, central Pacific. *Catena,* 44, 245-261.

Supplementary Table 2. Radiocarbon ages, Kandahalagalaa, Huvadhoo atoll, Maldives.

Lab code	Island sample location	Sample material	Elevation (msl, m)	Conventional age (yr B.P.)	Calibrated age range (95.4% probability) (cal. yr B.P.)	Mid-point of calibrated age range (cal. yr B.P.)
**Moat and reef samples**						
Wk-46719	KAND_PC2_55	Coral (*Porites*)	-2.57	3,255 ± 49	3,185 – 2,750	2,963
D-AMS 026822	KAND_PC1_70	Coral (*Acropora*)	-2.98	2,806 ± 33	2,677 – 2,234	2,433
Wk-46724	KAND_PC1_78	Coral sand	-3.06	3,145 ± 51	3,072 – 2,637	2,836
Wk-46723	KAND_PC1_84	Coral sand	-3.12	3,016 ± 35	2,872 – 2,454	2,683
Wk-46725	KAND_PC3_90	Coral sand	-2.75	1,783 ± 42	1,396 – 1,035	1,222
**Island samples**						
Wk40681	KAN_C3_140	*Halimeda*	0.61	2,026 ± 20	1,650 – 1,299	1,475
Wk40661	KAN_C3_320	*Halimeda*	-1.19	1,849 ± 20	1,465 – 1,121	1,293
Wk40666	KAN_C4_45	*Halimeda*	1.09	966 ± 20	602 - 296	449
Wk40664	KAN_C4_120	*Halimeda*	0.34	1,732 ± 20	1,321 - 994	1,156
Wk40665	KAN_C4_200	*Halimeda*	-0.45	3,277 ± 20	3,187 – 2,786	2,987
Wk40667	KAN_C6_40	*Halimeda*	1.25	1,134 ± 20	725 - 460	592
Wk40668	KAN_C6_130	*Halimeda*	0.35	1,826 ± 20	1,423 – 1,084	1,254
Wk40669	KAN_C6_215	*Halimeda*	-0.50	1,975 ± 20	1,591 – 1,266	1,429
Wk40670	KAN_C6_320	*Halimeda*	-1.55	1,800 ± 20	1,390 – 1,065	1,228
Wk42999	KAN_C6_320b	Bulk sand	-1.55	1,700 ± 29	1,293 - 961	1,127
Wk40671	KAN_C8_110	*Halimeda*	0.17	2,347 ± 20	2,049 – 1,658	1,853
Wk40672	KAN_C8_165	*Halimeda*	-0.38	1,919 ± 20	1,530 – 1,200	1,365
Wk40673	KAN_C9_100	*Halimeda*	0.45	1,707 ± 20	1,295 - 971	1,133
Wk42997	KAN_C9_100b	Bulk sand	0.45	1,198 ± 31	795 - 496	646
Wk40674	KAN_C9_170	*Halimeda*	-0.25	1,369 ± 20	952 - 651	802
Wk40675	KAN_C9_312	*Halimeda*	-1.67	838 ± 20	495 - 168	332
Wk42998	KAN_C9_312b	Bulk sand	-1.67	1,260 ± 20	867 - 547	707
Wk40676	KAN_C12_45	*Halimeda*	0.65	1,489 ± 20	1,093 - 739	916
Wk40659	KAN_C12_150	*Halimeda*	-0.20	1,994 ± 20	1,610 – 1,279	1,445
Wk40677	KAN_CH12_235	*Halimeda*	-0.85	2,321 ± 20	2,004 – 1,617	1,811
Wk40660	KAN_C12_300	*Halimeda*	-1.65	1,516 ± 20	1,130 - 770	950
Wk40302	KAN_C12_345	Coral stick	-2.08	2,455 ± 20	2,184 – 1,773	1,979
Wk40678	KAN_C13_150	*Halimeda*	-0.23	1,897 ± 20	1,509 – 1,179	1,344
Wk40679	KAN_C13_300	*Halimeda*	-1.73	2,373 ± 20	2,080 – 1,695	1,889
Wk40680	KAN_C13_350	*Halimeda*	-2.23	2,888 ± 20	2,712 – 2,342	2,527
Wk40663	KAN_C19_130	*Halimeda*	0.10	1,165 ± 20	754 - 484	619
Wk40662	KAN_C19_305	*Halimeda*	-1.65	1,096 ± 20	691 - 427	559
**Beachrock samples**						
D-AMS 036938	KAND_BR1_C1	Beachrock	0.79	1,688 ± 27	1,285 - 955	1,130
D-AMS 036939	KAND_BR1_C2	Beachrock	0.72	1,765 ± 23	1,362 – 1,035	1,205
D-AMS 036940	KAND_BR2	Beachrock	0.80	1,752 ± 25	1,347 – 1,015	1,193
D-AMS 036941	KAND_BR2_B	Beachrock	0.50	1,885 ± 22	1,505 – 1,170	1,328
D-AMS 036942	KAND_BR3	Beachrock	0.79	1,923 ± 22	1,535 – 1,205	1,366
D-AMS 036943	KAND_BR3A	Beachrock	0.79	1,970 ± 28	1,594 – 1,256	1,414
D-AMS 036944	KAND_BR4_UP	Beachrock	0.37	1,830 ± 24	1,436 – 1,090	1,271
D-AMS 036945	KAND_BR4_L	Beachrock	0.22	1,934 ± 25	1,550 – 1,215	1,377
D-AMS 036946	KAND_BR5	Beachrock	-0.29	1,838 ± 22	1,451 – 1,105	1,279
D-AMS 036947	KAND_BR6	Beachrock	0.30	1,062 ± 22	670 - 395	538
D-AMS 036948	KAND_BR7	Beachrock	-0.56	1,094 ± 23	690 - 424	562
D-AMS 036949	KAND-BR8	Beachrock	-0.36	1,052 ± 31	668 - 372	529

Note: Radiocarbon dates obtained from the Radiocarbon Dating Laboratory, University of Waikato (Wk), New Zealand and Direct-AMS, USA. Ages calibrated using OxCal version 4.4[52] with Marine 20 curve[53] and Delta-R (-46, 51) as best estimate for the central Indian Ocean.

Supplementary Table 3. Physical properties of beach rock outcrops on Kandahalagalaa, Huvadhoo Atoll, Maldives. Location and character of beachrocks shown on Fig. 3.

Number	BR Strike relative to north (deg)	Paleo beach exposure (deg)	BR- dip (slope, %)	Length (m)	Width (m)	Elevation max (m)	Max. dist. from shoreline (m)
Shoreline	113.7	24	10.3	-----	-----		0
BR-1	54.8	144	11.3	56	17.9	0.69	61
BR-2	66.3	156	14.0	21	2.9	0.80	20
BR-3	100.5	190	9.3	5	1.7	0.79	3
BR-4	61.6	151	1.3	23	3.8	0.37	25
BR-5	58.6	148	16	15	2.9	-0.29	18
BR-6	72.3	342	21.6	70	11.0	0.30	70
BR-7*	106.9	17	8.4	41	4.6	-0.56	57
BR-8*	106.4	16	14.2	27	9.1	-0.36	63

*Denotes beachrocks detached from contemporary shoreline and below mean sea level.

Supplementary Table 4. Calculated vegetated area of Kandahalagalaa Island, Huvadhoo atoll, 1969-2021 for the aerial images used in the analysis.

Year	Island area (m^2)
1969	54051.0
2005	56492.5
2006	56292.9
2009	54650.7
2011	54555.0
2014	55537.3
2016	56921.7
2017	57478.6
2018	57372.1
2019	57363.1
2020	56468.6
2021	56411.2
